# Features of Polycystic Ovary Syndrome in adolescence

**Published:** 2015

**Authors:** P Tsikouras, L Spyros, B Manav, S Zervoudis, C Poiana, T Nikolaos, P Petros, M Dimitraki, C Koukouli, G Galazios, GF von Tempelhoff

**Affiliations:** * Department of Obstetrics and Gynaecology, Democritus University of Thrace, Greece; ** Department of Obstetrics/Gynaecology, “Carol Davila” University of Medicine and Pharmacy, Bucharest, Romania; Rea Hospital Athens, Greece; *** Department of Endocrinology, “Carol Davila” University of Medicine and Pharmacy, Bucharest, Romania; **** Department of Obstetrics and Gynaecology, Clinicum Aschaffenburg, Teaching Hospital University of Würzburg, Germany

**Keywords:** Polycystic Ovary Syndrome, adolescence, puberty, anovulation, hyperandrogenism

## Abstract

**Rationale:** To elucidate the prepubertal risk factors associated with the development of Polycystic Ovary Syndrome (PCOS) and determine the special clinical manifestations of the syndrome in this transitional time of a woman’s life.

**Objective:** To propose therapeutic targets and regimens, not only to prevent the long-term complications of the syndrome, but also to improve the self-esteem of a young girl who matures into womanhood.

**Methods and Results:** A systematic review of literature was performed through electronic database searches (Pubmed, Medline and Embase). Studies published in English-language, peer-reviewed journals from 1996 to 2013 were included. The selected studies focused on the risk factors, the unique features and treatment options of the PCOS in puberty. The pathogenesis of the PCOS was hypothesized to be based on interactions between genetic and certain environmental factors. The diagnosis was usually difficult in young girls. The syndrome was related to a greater risk of future infertility, type II diabetes mellitus, the metabolic syndrome and cardiovascular disease. Early treatment was crucial to prevent the long-term complications of the syndrome, especially infertility and cardiovascular disease.

**Discussion:**The recognition of the early signs of PCOS during or even before adolescence is of great importance. It is essential to establish the correct diagnosis for PCOS and rule out other causes of androgen excess in young women with hyperandrogenism. The type of treatment applied should be considered on an individual basis.

**Abbreviations:** PCOS = Polycystic Ovary Syndrome

## Introduction

Polycystic ovary syndrome (PCOS) is a heterogeneous condition often associated with oligo anovulation, clinical or biochemical hyperandrogenism due to ovarian dysfunction. Ovarian dysfunction continues to be the pivotal feature that makes this syndrome the major cause of anovulatory associated infertility in developed countries [**1**,**2**].

The Polycystic Ovary Syndrome (PCOS) represents the most common endocrine disorder among women of reproductive age, with a mean incidence of 5-10% [**3**].

PCOS is defined by specific clinical, biochemical and ultrasonographic criteria [**4**]. Clinical manifestations include menstrual irregularities, signs of androgen excess and obesity [**5**].

It is characterized by endocrine and metabolic disorders. Although the clinical manifestations of the syndrome depend on the age of the woman, ovarian malfunction and hyperandrogenism are common features at any age.

The pathogenesis of PCOS is unknown; however, it is believed that the syndrome is the result of interactions between genetic and one or more environmental factors. Although the precise role of certain genes was not yet elucidated, a number of study findings are indicative of a genetic predisposition among family members [**6**,**7**]. Recently, the promoter -1031(T/ C) polymorphism in tumour necrosis factor-alpha was linked to PCOS [**8**,**9**].

According to the European Society for Human Reproduction and Embryology (ESHRE) and the American Society for Reproductive Medicine (ASRM) consensus on the diagnostic criteria of PCOS (2003), the syndrome is diagnosed when two of the following three criteria are present: (1) Anovulation or oligoovulation, (2) biochemical hyperandrogenemia or hyperandrogenism, (3) polycystic ovaries observed ultrasonographically [**5**]. However, in 2006, the Androgen Excess Society (AES) decided to exclude those women in whom there was no hyperandrogenemia or hyperandrogenism from the diagnosis of the syndrome. Thus, the currently accepted criteria include hyperandrogenism, anovulation and/ or polycystic ovaries as observed by ultrasonography and when other causes of hyperandrogenism (such as congenital adrenal hyperplasia or androgen-secretory tumours) and ovarian dysfunction (such as hyperprolactinaemia or thyroid gland impairment) have been excluded [**10**,**11**]. PCOS is also accompanied by a number of metabolic disorders, such as insulin resistance and hyperinsulinemia, dyslipidemia and obesity. However, the metabolic manifestations of the syndrome are not included in its criteria.

While the first signs of PCOS can be perceptible even during childhood, the unique features of the syndrome in puberty are not yet clear [**12**]. Despite these difficulties, an early diagnosis of PCOS is of great importance, since its presence is related to a greater risk of future infertility, type II diabetes mellitus, metabolic syndrome and cardiovascular disease. The diagnosis of PCOS in puberty can be difficult, as anovulation is very common in young girls (1/ 2 menstrual cycles are anovulatory in the first two years after menarche), while ultrasound display of multiple follicles is also a fairly common finding during puberty [**12**]. Thus, biochemical hyperandrogenemia or hyperandrogenism with hair excess are at present the main findings indicating diagnosis of the syndrome at this age. Besides that, there are some studies that claimed that there is a strong association between PCOS and certain types of congenital uterine anomalies suggesting that there is a developmental defect that could be found in early ages even before adolescence [**13**,**14**]. Moreover, other studies claimed that there are some inflammatory markers closely related to the PCOS syndrome that could explain the pathogenesis of the syndrome in short time [**15**].

The aim of this review is to elucidate the prepubertal risk factors associated with the development of the syndrome, the unique diagnostic criteria and the special clinical manifestations of PCOS in adolescence. Therapeutic targets and regimens suitable and safe for this stage of a woman’s life are also proposed.

## Methods

**Sources**

A systematic review of the literature cited in Pubmed, Medline and Embase, covering a period of 15 years (1996-2014), was performed. The terms Polycystic Ovary Syndrome, adolescence, puberty, anovulation, hyperandrogenism, metabolic disorders, diagnosis, risk factors and treatment were used in various combinations during the search. All reviewed studies were published in English language.

**Study selection**

Twenty-four studies, both original and reviews, published in peer-reviewed journals, were included. The authors considered adolescent PCOS as a distinct clinical and biochemical entity. Thus, the selected studies focused mainly on the risk factors, the unique diagnostic criteria and clinical manifestations, and the therapeutic options of PCOS specifically in puberty rather than in women of reproductive age.

## Results

**Prepubertal risk factors implicated in the development of PCOS**

Although PCOS is traditionally considered a disorder afflicting women of reproductive age, in fact it affects the entire life of the woman, starting from intrauterine development and going through menopause and beyond into later life. The clinical manifestations of the symptoms can be observed starting around menarche; however, there are signs that may alert us to its earlier onset.

The clinical appearance includes the following signs: irregular menses/ amenorrhea, oligo/ anovulation, and elevated circulating concentrations of androgens and/ or features of hyperandrogenism. In addition, approximately 75% of women with PCOS have insulin resistance and hyperinsulinemia, and about 50% have elevated levels of circulating luteinizing hormone (LH) [**16**,**17**]. Women most commonly seek counseling or treatment because of infertility due to chronic anovulation. Insulin resistance accompanied by compensatory hyperinsulinemia constitutes another major biochemical feature of polycystic ovary syndrome, which leads to early luteinizing hormone sensitivity of the follicle and to stimulation of both ovarian and adrenal androgen production [**18**,**19**]. The woman with this syndrome usually visits the gynecologist with menstrual dysfunction and complaints secondary to hyperandrogenism or unsuccessful reproduction.

Congenital masculinization syndromes (mainly congenital adrenal hyperplasia), premature menarche or premature pubarche, low birth weight for gestational age, obesity and the metabolic syndrome have been identified as independent prepubertal risk factors for the development of PCOS [**20**].

PCOS is common in girls with Congenital Adrenal Hyperplasia (CAH). The diagnosis of PCOS against a background of a pre-existing CAH is likely when anovulation is observed, combined with elevated testosterone levels, in women with well-regulated adrenal disease. PCOS is diagnosed in about half of the women with the classical type of CAH and in one fifth of the women with the non-classical type. Non-classical CAH in particular is most common during puberty.

Insulin resistance is also a common feature of non-obese females with non-classical CAH before treatment with glucocorticoids. Ineffective treatment of CAH leads to elevated LH levels, polycystic ovaries and infertility. Recent studies have reported that intrauterine exposure to androgens affects the progesterone’s normal inhibition of LH secretion. Exposure of female Rhesus monkeys to androgens during early gestation results in PCOS development [**20**].

Ibanez et al. reported that premature adrenarche, premature pubarche and exaggerated ovarian androgen synthesis are linked to PCOS [**21**,**22**]. Treatment with GnRH-agonists leads to an overreaction of LH and a dramatic increase in adrenal androgens, such as 17a-hydroxy-progesterone. Therefore, girls thus affected are at greater risk of developing PCOS. Premature pubarche also represents an independent risk factor in 15-20% of young females for PCOS in later adolescence. The syndrome should be anticipated to occur in any girl with premature pubarche, obesity and persistent oligomenorrhea [**23**]. According to Ibanez et al. “low birth weight for gestational age” is also associated with premature pubarche in later childhood and thus with PCOS [**24**].

McCartney et al. disclosed the fact that obesity predisposes to the development of insulin resistance, hyperinsulinemia and PCOS [**25**]. Adolescent obesity results in a subclinical increase in testosterone levels. Hyperandrogenemia inhibits the negative feedback of progesterone, usually by suppressing LH secretion. Thus, pubertal obesity could lead to elevated LH levels and the development of PCOS in the young girl [**25**]. 

Over the past few decades, there has been a striking increase in the prevalence of obesity among adolescents. Today, almost 16% of girls between the ages of 12 to 19 are significantly overweight and 32% are at risk [**26**]. Given the association of PCOS with obesity and the current trend of rising incidence of obesity, an increase in the prevalence of the syndrome in the near future seems inevitable [**27**]. Finally, the metabolic syndrome is related to insulin resistance and may thus carry a higher risk for PCOS [**19**].

**Clinical manifestations and laboratory findings of PCOS in adolescence (**Tables 1**,**2**)**

**Table 1:** Leading symptoms of PCOS in adolescence

Study	Finding	%
Christensen SB et al. 2013 [**45**]	Anovulation	64.7%
Gambineri A et al. 2013 [**46**]	Hirsutism	16.7%%
Gambineri A et al. 2013 [**46**]	Acne	29%
Christensen SB et al. 2013 [**45**]	Androgenic alopecia	4.2%%
Gambineri A et al. 2013 [**46**]	Obesity	50%
Christensen SB et al. 2013 [**45**]	Insulin resistance	62.8%

**Table 2:** Main laboratory features of PCOS in adolescence [**17**,**18**].

Hormones	**	Metabolic markers	**
Testosterone	*high/ normal*	A1-apolipoprotein	*low*
Free Androgen Index (FAI)	*high*	Cholesterol	*normal/ high*
Sex Hormone Binding Globulin (SHBG)	*low*	HDL/ LDL cholesterol ratio	*low*
D4-Androstendione	*normal/ high*	Triglycerides	*normal/ high*
DHEAS	*normal/ high*	Insulin	*high/ normal*
LH	*normal/ high*	Hba1c	*high/ normal*
LH/FSH ratio	*normal/ high*		**

During the first years after menarche, many adolescent girls experience anovulation. The most common manifestations are primary amenorrhea (lack of menarche occurring after the age of 15), arrheomenorrhea (less than 8 menstruations per year) and secondary amenorrhea (lack of menstruation for more than 6 months). Dysfunctional bleeding of the uterus is also common (bleeding occurring at intervals of less than 21 days, or lasting at least 7 days).

Hyperandrogenism is fairly common in adolescent girls, particularly those with PCOS. The leading symptom is the development of male type hair growth, or hirsutism. It is measured by the Ferriman-Gallwey scale, which represents the extent of hair growth in androgen-dependent regions of the body. Hyperandrogenism should also be considered in cases of resistant acne developing before menarche. Other features are alopecia, hyperhidrosis and seborrhea.

Due to an increasingly unhealthy lifestyle, growing numbers of adolescent girls are at risk of becoming obese and thus develop a metabolic syndrome. Obesity is found in 50% of the patients with PCOS and is linked to insulin resistance, the metabolic syndrome and cardiovascular complications. It is notable that a large number of young girls with PCOS have a normal body weight and will not develop clinical symptoms of the syndrome until they become overweight. According to Leibel et al., the frequency of the metabolic syndrome in PCOS patients is of approx. 25% [**28**].

**Imaging**

The established ultrasonographic criteria for the diagnosis of polycystic ovaries include the presence of enlarged ovaries (ovarian volume >10 cm3), with multiple follicles (>11 in each ovary) 2-9 mm in size. However, ovaries with these characteristics may be observed in normal adolescents up to two years after menarche. Polycystic ovarian appearance of this nature may also be observed in normal adult women. The incidence of polycystic ovarian appearance is smaller in adolescents, in whom the transabdominal ultrasound is generally used, as compared to adults who are usually examined by transvaginal ultrasound. It has not been elucidated yet whether this difference is real or is due to the different imaging capacity of the two methods [**6**,**29**].

**Diagnosis and differential diagnosis**

In young women who are suspected to have the PCOS, serum androgens levels (total and free testosterone, Free Androgen Index, DHEAS, D4-Androstendione, 17a-Hydroxy-Progesterone) and SHBG need to be determined. If hyperandrogenemia is detected, a dexamethasone suppression test and the ACTH-test (cosyntropin stimulation test) should be performed in order to determine the origin of the androgens, ovarian or adrenal, and to exclude such disorders as late onset of the adrenogenital syndrome. 

Moreover, the differential diagnosis of PCOS in puberty includes disorders of steroidogenesis, resistance in glucocorticoids, acromegaly, the Cushing syndrome, hyperprolactinemia, insulin resistance, hypothyroidism, androgen-secretory tumours, medication (valproic acid, anabolic steroids) and sex differentiation disorders. 

An ultrasound examination of the ovaries should be performed to exclude an androgen-secretory tumour and to determine the possibility of polycystic ovarian morphology. Additionally, a thorough hormonal evaluation of the teenager with hyperandrogenemia is crucial, including prolactin, serum cortisol, insulin-like growth factor I (IGF-I), TSH and free T4. 

After the diagnosis of the PCOS is established, the young patient should be subjected to an oral glucose tolerance test (OGTT) to exclude impaired glucose tolerance [**30**,**31**]. If the increased androgen levels cannot be suppressed by dexamethasone, an MRI is mandatory for adrenal depiction to rule out an adrenal tumour [**32**].

However, in about 8% of the cases of hyperandrogenemia, there is no obvious etiology and it is considered to be primary.

**Treatment**

The treatment of PCOS in adolescent girls should aim to achieve ovulation, normalize the menstrual cycle, reduce and if possible eliminate hirsutism and acne, achieve weight loss, as well as to treat hyperlipidemia and hyperglycaemia in order to lower the risk of cardiovascular disease. Most protocols in puberty also aim to reduce the androgen excess.

However, most importantly, a healthy diet combined with exercise should be proposed as the number one priority, especially in overweight adolescent women. Losing weight not only contributes to the prevention of the clinical manifestations of the PCOS, but also improves the young girl’s self-esteem [**33**]. Similarly, the use of cosmetics as well as hair removal, used in conjunction with androgen suppression therapy, could be effective in the treatment of hirsutism. 

Oral contraceptives are the basis of hormone therapy, as they contribute to the reduction of hyperandrogenemia, hirsutism and acne [**34**-**36**]. A combination of 35mg ethinyl estradiol and 2 mg cyproterone acetate is usually administered. The latter is an antiandrogen, particularly effective in the reduction of testosterone and D4-androstendione and the normalization of the LH/ FSH ratio. However, it seems to have a negative effect on the lipid profile, causing a significant increase of triglyceride levels [**37**]. Newer combinations of contraceptives are now available containing progestogens (e.g. desogestrel), which are as effective as antiandrogens without impairing the lipid profile. Finally, a combination of 30mg ethinyl estradiol and drospirenone is also advisable. Its main advantage is that it does not cause weight gain due to its mild diuretic effects.

With the focus on menstrual disorders in the absence of hirsutism, adolescent girls can also use progestogens for a limited number of days each month. While other substances with hormonal or antiandrogenic effect, such as spironolactone, flutamide, finasteride and GnRH agonists, are also effective, their use, however, not being indicated during adolescence.

Metformin, aimed to manage insulin resistance, is widely used in the treatment of PCOS, in daily doses ranging from 500 mg to 2000 mg. It has been shown to reduce hyperandrogenemia by increasing the levels of SHBG. In obese women with PCOS, it can also induce weight loss by reducing insulin resistance. Lastly, metformin is beneficial in adolescent girls with PCOS with regard to ovulation, the normalization of the menstrual cycle and even loss of weight [**38**,**39**]. 

Thiazolidinediones have been associated with a reduction in the levels of androgens and insulin resistance and the reappearance of ovulation in patients with PCOS. In a number of studies, rosiglitazone and pioglitazone are reported to be effective in restoring normal menstrual cycles and reducing insulin levels in women with PCOS [**40**,**41**].

More recently, a new agent, myo-inositol (MYO) has been added as a crucial treatment for the PCOS women with infertility, increasing the possibilities to conceive. Myo-inositol is a vitamin B-like substance that can function as the basis of a number of signalling and secondary messenger molecules in insulin signalling pathways. Myo-inositol seems to correct the mal-functioning insulin pathways and reduce the signs and symptoms of insulin resistance [**42**].

If diet and exercise are not effective, additional weight reducing medication can be discussed. Orlistat inhibits the intestinal absorption of fat and sibutramine acts directly on the appetite center of the brain. Both cause additional weight loss of 3% and 8.5%, respectively [**43**,**44**]. However, there is not yet sufficient evidence regarding the safety of these drugs in adolescence [**43**].

## Discussion

The pathogenesis of PCOS is hypothesized to be based on interactions between genetic and certain environmental factors. Thus, the possibility of a young girl developing PCOS should always be considered when the syndrome or simply polycystic ovaries, are present in the adolescent and her mother or when obesity, diabetes mellitus or other causes of insulin resistance occur in either of the parents. The recognition of the early signs of PCOS during or even before adolescence is of great importance. It is important to establish the correct diagnosis for PCOS and rule out other causes of androgen excess in young women with hyperandrogenism. Early treatment is crucial to prevent the long-term complications of the syndrome, and most particularly infertility and cardiovascular disease. 

Further research is necessary to elucidate the mechanisms by which PCOS disrupts the endocrine harmony of the young female body in that crucial, transitional period of a woman’s life.
